# Familial associations between ANCA-associated vasculitis and other immune-mediated inflammatory diseases: a case control study

**DOI:** 10.1007/s00296-026-06259-1

**Published:** 2026-07-24

**Authors:** Justin Chong Meng Chua, Kevan Roy Polkinghorne, Samar Ojaimi, Jessica Ryan, Arthur Richard Kitching

**Affiliations:** 1 Department of Medicine, Monash Medical Centre, Monash University, 246 Clayton Rd, Clayton, VIC 3168 Australia; 2 Department of Nephrology, Monash Health, Clayton, VIC Australia; 3 Centre for Inflammatory Diseases, Monash University, Clayton, VIC Australia; 4 School of Public Health and Preventive Medicine, Monash University, Melbourne, VIC Australia; 5 Monash Health Pathology, Monash Health, Clayton, VIC Australia; 6 Monash Lung Sleep Allergy Immunology, Monash Health, Clayton, VIC Australia; 7 Monash Infectious Diseases, Monash Health, Clayton, VIC Australia; 8 Department of Paediatric Nephrology, Monash Children’s Hospital, Clayton, VIC Australia

**Keywords:** Autoimmune diseases, Anti-neutrophil cytoplasmic antibody-associated vasculitis, Risk factors, Granulomatosis with polyangiitis, Microscopic polyangiitis, Genetic association studies

## Abstract

This study assesses the relationship between ANCA-associated vasculitis and immune-mediated inflammatory diseases (IMIDs) in first-degree relatives. People with AAV were matched 1-to-1 with control participants with no IMID in a case control study. Participants were asked whether their first-degree relatives had IMID. The likelihood of a positive family history was compared between cases and controls using conditional logistic regression. Additionally, the risk of first-degree relatives of cases having IMID was explored using generalised estimating equations. 145 AAV cases were matched with 145 controls. A family history of IMID was reported by 46.2% of those with AAV and 16.6% of controls. After adjustment for participant age and number of known family members, AAV cases were seven-times more likely to have a positive family history compared to controls (OR 7.25, 95% C.I. 3.27–16.09). 12% of first-degree relatives of AAV participants had an IMID compared to 3.5% of first-degree relatives of control participants. First-degree relatives of AAV participants were four-times more likely to have an IMID compared to a relative of a control (OR 4.03, 95% C.I. 2.46–6.62). Female first-degree relatives had double the risk of having an IMID, compared to male first-degree relatives. A history of IMID in first-degree family members is associated with an increased chance of a person developing AAV. Conversely, the presence of AAV in a first-degree relative is associated with having an increased chance of having an IMID. This implies a shared familial susceptibility between AAV and these diseases.

**Supplementary Information:**

The online version contains supplementary material available at 10.1007/s00296-026-06259-1.

## Introduction

Anti-neutrophil cytoplasmic antibody (ANCA)-associated vasculitis (AAV) involves autoimmune inflammation of small blood vessels that is mediated, at least in part, by circulating antibodies to one of two neutrophil granule proteins, proteinase-3 (PR3) or myeloperoxidase (MPO) [1]. AAV includes three main syndromic presentations: microscopic polyangiitis (MPA), granulomatosis with polyangiitis (GPA) and eosinophilic granulomatosis with polyangiitis (EGPA). The causes of AAV are not well understood but AAV is likely to result from complex interactions between genetic and environmental factors [2, 3].

Several genome wide association studies (GWAS) have defined genetic polymorphisms that increase the risk of developing AAV [4–6]. Some of these polymorphisms are in genes linked mechanistically and specifically to AAV. For example, in people with PR3-AAV, polymorphisms impacting the expression of PR3 and its inhibitor, alpha-1 anti-trypsin, modify the risk of developing disease [4]. However, genetic polymorphisms associated with AAV also involve loci impacting common immune pathways such as human leukocyte antigen (HLA) complexes and T cell activity, and have been associated with other immune diseases [3, 7–10].

While familial aggregation of systemic lupus erythematous and other immune-mediated inflammatory diseases has been defined [11–13], there is a paucity of studies examining the relationship of AAV and immune-mediated inflammatory diseases in families. One registry-based population study from Sweden found a modestly increased risk of immune-mediated inflammatory diseases in first-degree relatives of GPA patients, compared with healthy controls [14].

We therefore investigated whether immune-mediated inflammatory diseases in first-degree relatives is a “risk factor” for AAV. We hypothesised that there would be an association between having AAV and having first-degree relatives with immune-mediated inflammatory diseases. In a related but different aim, we also assessed the likelihood of individual first-degree relatives of AAV patients having an immune-mediated inflammatory disease, compared with relatives of those without AAV. We hypothesised that there would be an increased likelihood of first-degree relatives of AAV patients having immune-mediated inflammatory diseases.

## Methods

### Study design and participants

We conducted a case-control study at Monash Health, a large Australian multi-site tertiary healthcare network with a dedicated vasculitis clinic and large nephrology service. Recruitment occurred between November 2023 and June 2024. To recruit cases and controls, English-speaking adult patients (age *≥* 18 years) were contacted by email, in-person at clinics, or by telephone after receiving an introductory email describing the study. Participants needed to have at least one first-degree relative known to them to participate. This study was performed in line with the principles of the Declaration of Helsinki and was approved by the Monash Health Human Research Ethics Committee (RES-23-0000-391 A) on 19th September 2023. Informed consent was obtained from all subjects and recorded electronically before commencement of the survey.

### Study cases

Cases were defined as patients with a clinical diagnosis of AAV with MPA or GPA, which included assessment of clinical presentation, the presence of MPO and PR3-ANCA, radiological and other laboratory tests, and histopathology. We also classified cases as GPA or MPA according to 2022 American College of Rheumatology/European Alliance of Associations for Rheumatology classification criteria [15, 16].

Recruitment occurred from three speciality outpatient clinics – a dedicated vasculitis clinic (predominant source), a dialysis clinic and kidney transplant clinic. Patients with EGPA were excluded due to the EGPA’s greater differences in ANCA profile, clinical presentation and pathology compared to MPA and GPA. To prevent double counting families with more than one AAV case, only one AAV case per family participated. People with AAV who had additional immune-mediated inflammatory diseases were included, though this was not analysed separately.

### Study controls

Control subjects were recruited from nephrology clinics. All controls had kidney disease, either having chronic kidney disease not requiring dialysis, undergoing dialysis or received a kidney transplant. Exclusion criteria for controls included any immune-mediated inflammatory disease (including those related to kidney disease) and kidney disease of unknown cause. Additionally, controls could not have a first-degree relative with AAV.

Each case was matched 1:1 without replacement to a control by biological sex and age (± five-years).

### Assessment of immune-mediated inflammatory disease history in participants and first-degree relatives

Participants were provided with direction on completing the survey in an information sheet which contained a non-exhaustive list of immune-mediated inflammatory diseases (Supplementary Figure S1). As there is no consensus definition of immune-mediated inflammatory disease, nor a standardised list of these diseases, the listing and nomenclature of immune diseases was determined by investigators (including a clinical immunologist [SO]), after referring to relevant sources [17, 18]. This information sheet was emailed to each participant, with participants also given a paper version if spoken to in-person by investigators. Participants completed the questionnaire either online (instructions provided on the webpage), or by interview with investigators in-person or by telephone, with verbal prompts provided that were similar to the instructions on the webpage.

Participants were asked a series of questions including their date-of-birth, whether they had been diagnosed with AAV or another immune-mediated inflammatory disease, and the number of their first-degree relatives (parents, siblings and children). For each relative identified, they were asked their biological sex and whether they had knowledge about their medical conditions (‘known’ relatives). Only ‘known’ first-degree relatives were included in the analyses. For each ‘known’ relative, participants were asked whether the relative had one or more immune-mediated inflammatory disease(s), though they were only assessed for having a ‘positive’ history as having multiple immune-mediated inflammatory diseases was not included as a variable in the analyses. The participants were also asked whether they thought the identified conditions were ‘definite’ or ‘probable’. For conditions listed as ‘probable’ (*n* = 21 conditions), participants were contacted with a follow-up phone call to clarify details of the relative’s disease. The resultant information was assessed by a clinical immunologist [SO] blinded to the participant’s group, who adjudicated on whether the condition was an immune-mediated inflammatory disease.

Medical records of AAV participants were reviewed to record their ANCA-subtype and organ systems affected by AAV. Records of controls were reviewed to check the cause of their kidney disease and to ensure they did not have immune-mediated inflammatory disease.

### Primary and secondary outcomes

The primary outcome compared the *risk of having a positive “family history”* (having at least one first-degree relative with one or more immune-mediated inflammatory disease) in cases, compared to control participants.

The secondary outcome assessed the *risk of individual first-degree relatives of cases having an immune-mediated inflammatory disease*, compared to first-degree relatives of control participants.

### Data management

Study data were collected and managed using the Research Electronic Data Capture (REDCap) tool hosted and managed by the Monash University’s Helix Data Platform [19, 20]. Participants either directly entered their information into REDCap via a survey link or had their data inputted by an investigator during the interview.

### Statistical analysis

All statistical analyses were performed using Stata (StataCorp, Texas, USA).

The matching of cases and controls was conducted using Stata. Baseline characteristics between cases and controls were compared using Chi-squared tests for categorical variables and t-tests and Mann-Whitney U tests for continuous variables.

For the primary outcome, conditional logistic regression models were used to assess the likelihood of a positive family history of immune-mediated inflammatory disease in cases compared to controls. All models included participant age and the number of first-degree relatives known to participants.

For the secondary outcome, to assess the risk of individual first-degree relatives of AAV participants having an immune mediated inflammatory disease, generalised estimating equations were used to account for the non-independence of multiple first-degree relatives within the same family [21, 22]. All models included participant age, the generational relationship of the first-degree relative to the participant (using children as the reference group) and the biological sex of first-degree relatives.

A power calculation was performed assuming 20% of controls will have at least one family member with immune-mediated inflammatory disease (~ 7% lifetime chance of immune-mediated inflammatory disease with mean of three first degree relatives) [23], and the OR is 2.5 in AAV cases. A total sample size of 190 (95:95) participants was required to achieve a power of 0.8 with α = 0.05 and β (Type II error) = 0.20.

We reported this study in accordance with the Strengthening the Reporting of Observational Studies in Epidemiology (STROBE) guidelines for case control studies [24] (Supplementary Table S1).

## Results

### Participant characteristics

**Fig. 1:**
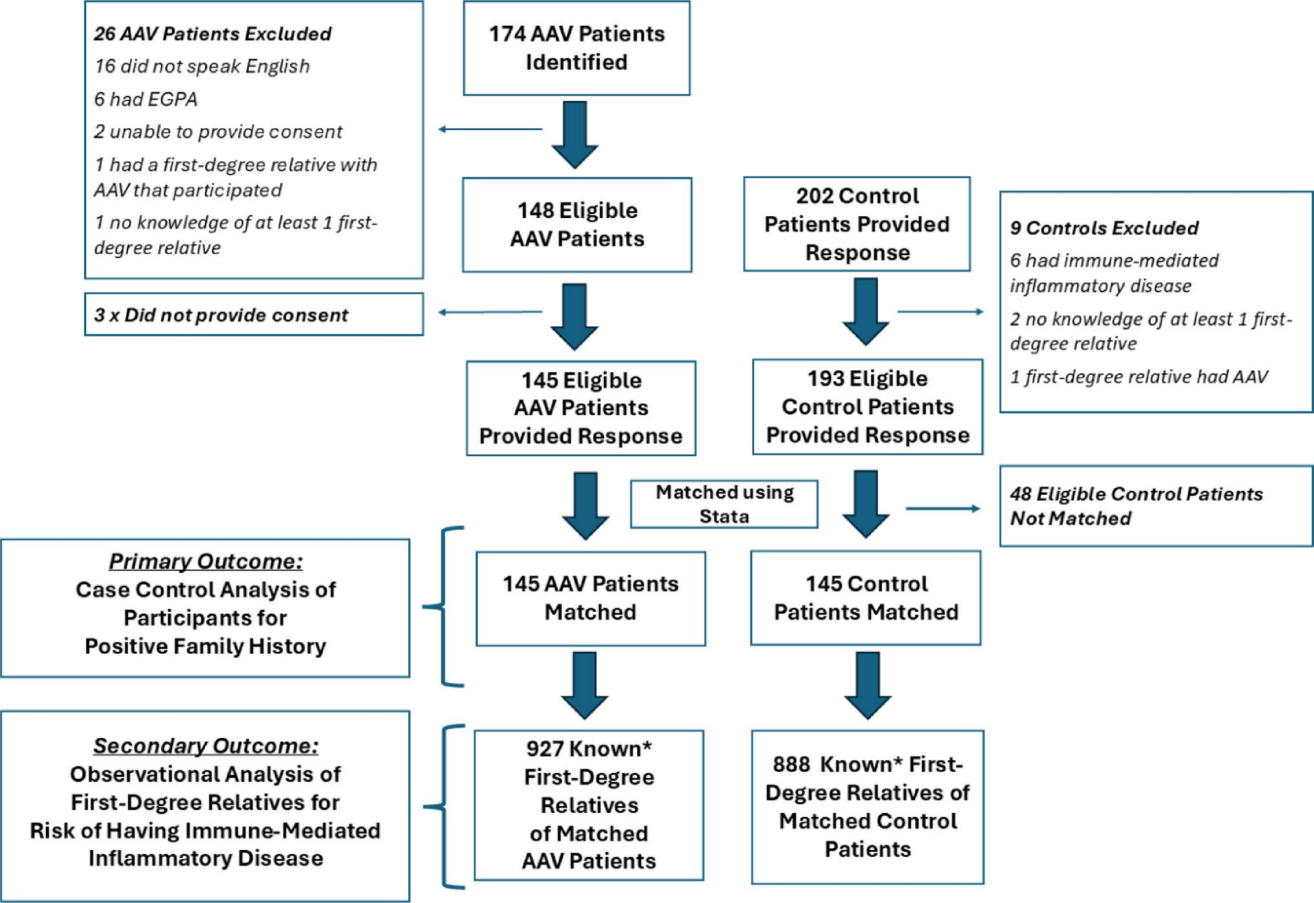
Recruiting and matching of ANCA-associated vasculitis (AAV) and control patients and subsequent analysis. *Known = medical history known to the participant

The participant recruitment and matching process is demonstrated in Fig. 1. From a total of 174 AAV patients identified in outpatient clinics, 148 were eligible for the study, of whom 145 participated (98.0%). There were 202 control participant responses, of which nine were excluded (6 had other immune-mediated inflammatory diseases, 2 had no knowledge of at least one first-degree relative, 1 had a first-degree relative with AAV), leaving 193 eligible control participant responses for matching. All responding 145 AAV participants (cases) were matched with 145 control participants, leaving 48 control participants unmatched.

The characteristics of the cases and controls including their number of first-degree relatives are detailed in Table 1. Case and control participants were well matched on age (mean age of 62.4 and 62.6 years respectively) with 79 females (54.5%) in each group. Of the cases, 53.8% were MPO-ANCA + and 40.7% were PR3-ANCA+, with the kidneys (77.2%), lungs (50.3%) and ear, nose and throat (45.5%) being the most frequently affected tissues. Of the controls, the most common contributing causes of kidney disease were hypertension (60.7%), diabetes mellitus (not type 1) (41.4%) and renovascular disease (35.2%).

Cases and controls had similar proportions of first-degree relatives whose medical history was known to them (93.9% and 93.6% respectively), with the same median number of known first-degree relatives of 6 (IQR 4–8). There were a similar number of known parents, siblings and children per participant in each group.

**Table 1:** Characteristics of matched case (AAV) and control participants and their number of first-degree relatives

	Cases (AAV Participants)	Controls
Number of matched participants	145	145
Demographics		
Female	79 (54.5%)	79 (54.5%)
Age (years), mean (SD)	62.4 ± 16.5	62.6 ± 16.8
ANCA subtype		
MPO-ANCA+	78 (53.8%)	N/A
PR3-ANCA+	59 (40.7%)	
ANCA Negative	7 (4.8%)	
Both MPO- and PR3-ANCA+	1 (0.7%)	
AAV classification^#^		
Microscopic polyangiitis (MPA)	78 (53.8%)	N/A
Granulomatosis with polyangiitis (GPA)	67 (46.2%)	N/A
AAV organ and tissue involvement		
Kidneys	112 (77.2%)	N/A
Lungs	73 (50.3%)	
Ear, nose and throat	66 (45.5%)	
Musculoskeletal/joints	44 (30.3%)	
Cutaneous	32 (22.1%)	
Ocular	17 (11.7%)	
Peripheral nervous system	14 (9.7%)	
Trachea	11 (7.6%)	
Gastrointestinal	5 (3.4%)	
Central nervous system	1 (0.7%)	
Cardiac	1 (0.7%)	
Underlying causes of kidney disease in controls^*^		
Hypertension	N/A	88 (60.7%)
Diabetes mellitus (not type 1)		60 (41.4%)
Renovascular disease		51 (35.2%)
Single kidney (incl. nephrectomies)		16 (11.0%)
Cystic kidney disease (incl. polycystic kidneys)		12 (8.3%)
Cardio-renal		11 (7.6%)
Obstruction/renal stones		10 (6.9%)
Acute kidney disease for monitoring		6 (4.1%)
Reflux Nephropathy		6 (4.1%)
NSAID Use		4 (2.8%)
Lithium Nephropathy		2 (1.4%)
Other		5 (3.4%)
Number (%) of participants with one or more immune-mediated inflammatory disease in addition to AAV	38 (26.2%)	N/A
First-degree relatives		
Total number	987	949
Total known^b^ (and % of total group)	927 (93.9%)	888 (93.6%)
Known female (% of total known in group)	447 (48.2%)	445 (50.1%)
Known first-degree relatives by generation (known (%)/total number)		
Parents	276 (95.2%)/290	272 (93.8%)/290
Siblings	377 (91.5%)/412	353 (91.2%)/387
Children	274 (96.1%)/285	263 (96.7%)/272
Median (IQR) first-degree relatives known per participant		
Number known per participant	6 (4–8)	6 (4–8)
Parents	2 (2–2)	2 (2–2)
Siblings	2 (1–3)	2 (1–3)
Children	2 (1–3)	2 (1–3)

Known = Medical history known to the respondent. AAV = ANCA-associated vasculitis; ANCA = Anti-Neutrophil Cytoplasmic Antibodies; FDR = first-degree relative(s); IQR = interquartile range; MPO = myeloperoxidase; PR3 = proteinase 3; SD = standard deviation

* Control participants could have more than one reason for their kidney disease

###  Participants' family history of immune-mediated inflammatory diseases 

A summary of the proportion of cases and controls having a family history of immune-mediated inflammatory diseases, amongst first-degree relatives, is presented in Table 2. A positive family history (one or more first-degree relatives having at least one disease) was reported by 67 (46.2%) cases and 24 (16.6%) controls (*p* < 0.001). Cases with PR3-AAV and MPO-AAV had a similar proportion with a positive family history (44.1% and 47.4% respectively).

**Table 2:** Family history of immune-mediated inflammatory diseases in case and control participants

	Cases, *n* = 145 (AAV Participants)		Controls, *n* = 145	*p*-value
Participants with one or more known first-degree relative(s) with immune mediated inflammatory disease(s) [‘positive family history’], *n* (%)	67 (46.2%)		24 (16.6%)	< 0.001
Number of known first-degree relative(s) with immune mediated inflammatory disease(s)				< 0.001
Zero first-degree relatives	78 (53.8%)		121 (83.4%)	
One first-degree relative	40 (27.6%)		18 (12.4%)	
Two first-degree relatives	15 (10.3%)		5 (3.5%)	
Three first-degree relatives	8 (5.5%)		1 (0.7%)	
Four first-degree relatives	3 (2.1%)		0 (0%)	
Five first-degree relatives	1 (0.7%)		0 (0%)	
Known first-degree relatives with immune mediated inflammatory disease(s) per participant, mean (SD)	0.8 (± 1.1)		0.2 (± 0.5)	< 0.001

Known = Medical history known to the respondent; AAV = ANCA-associated vasculitis; SD = standard deviation

### Immune-mediated inflammatory diseases in first-degree relatives

Table 3 presents summary data on the disease types and number of affected first-degree relatives of cases and controls. The total number of first-degree relatives of cases with an immune-mediated inflammatory disease was 111 (12.0% of known first-degree relatives) compared to 31 (3.5% of known first-degree relatives of controls).

Supplementary Table S2 presents the immune-mediated inflammatory diseases reported and the number of AAV cases and first-degree relatives of cases and controls having these diseases. The most common types in first-degree relatives of cases were musculoskeletal and connective tissue (4.2% of known first-degree relatives), endocrine (3.9%), cutaneous (1.6%) and gastrointestinal diseases (1.1%). This compares to the first-degree relatives of control participants, with the most common type also being endocrine (1.0% of known first-degree relatives) and musculoskeletal and connective tissue (0.8%), though at much lower percentages. Only one of the cases had a first-degree relative with AAV.

**Table 3:** Immune-mediated inflammatory diseases in first-degree relatives of case and control participants

	Cases (AAV participants)		Controls	*p*-value
Total number (and percentage) of known first-degree relatives having immune-mediated inflammatory disease(s)	111 of 927 (12.0%)		31 of 888 (3.5%)	< 0.001
Number of known first-degree relatives (and percentage of that generation) with immune-mediated inflammatory disease(s)				
Parents	42 of 276 (15.2%)		12 of 272 (4.4%)	< 0.001
Sibling	47 of 377 (12.5%)		12 of 353 (3.4%)	< 0.001
Children	22 of 274 (8.0%)		7 of 263 (2.7%)	0.006
Disease types in known first-degree relatives (number and % of the total known first-degree relatives) ^a^				
Musculoskeletal and connective tissue	39 (4.2%)		7 (0.8%)	< 0.001
Endocrine	36 (3.9%)		9 (1.0%)	< 0.001
Cutaneous	15 (1.6%)		3 (0.3%)	0.006
Gastrointestinal	10 (1.1%)		2 (0.2%)	0.025
Vasculitis	7 (0.8%)		1 (0.1%)	0.049
Neurological	3 (0.3%)		4 (0.5%)	0.663
Haematological	3 (0.3%)		4 (0.5%)	0.663
Kidney	1 (0.1%)		1 (0.1%)	0.976
Other Immune	3 (0.3%)		0 (0%)	0.090

Known = Medical history known to the respondent; AAV = ANCA-associated vasculitis

### Primary outcome: likelihood of cases having a positive family history of immune-mediated inflammatory disease compared to controls

**Fig. 2:**
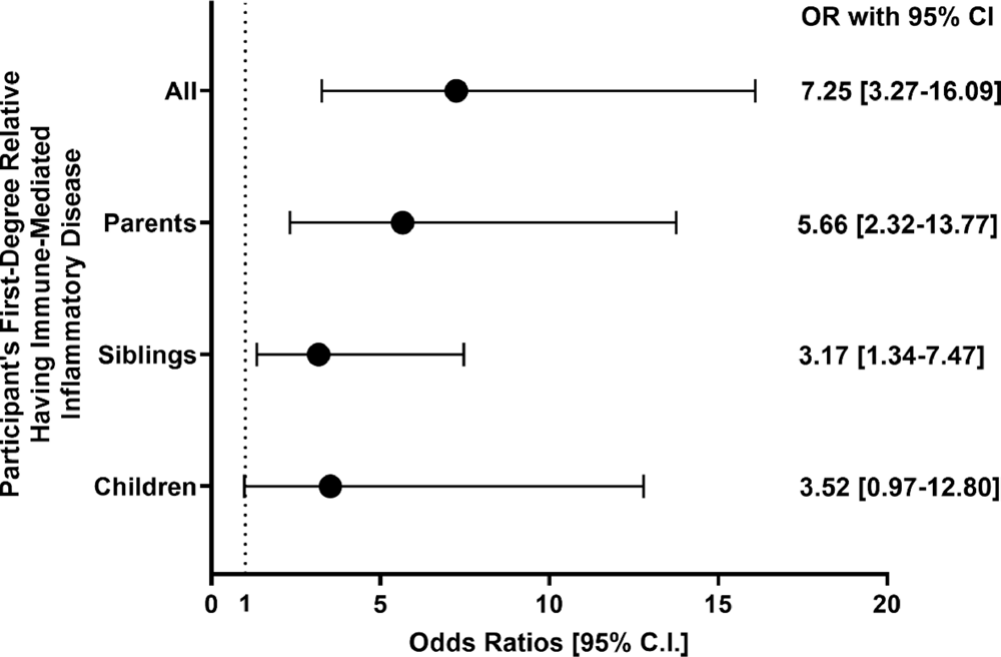
Relationship between participants having a family history of immune-mediated inflammatory diseases and participants having AAV. AAV = ANCA-associated vasculitis

Figure 2 presents the results of the primary outcome analysis. Cases were more than seven-times more likely to have a positive family history of immune-mediated inflammatory disease compared to controls (Odds Ratio (OR) 7.25, 95% C.I. 3.27–16.09). The risk of a positive family history stratified according to generational relationships of the first-degree relative to the participant (parents, siblings and children), is also presented in Fig. 2. Parents and siblings of cases were more likely to contribute to a positive family history (OR 5.66, 95% C.I. 2.32–13.77 and OR 3.17, 95% C.I. 1.34–7.47 respectively), while the risk due to children was uncertain (OR 3.52, 95% C.I. 0.97–12.80).

### Secondary outcome: risk of first-degree relatives of participants with AAV having an immune-mediated inflammatory disease

**Fig. 3:**
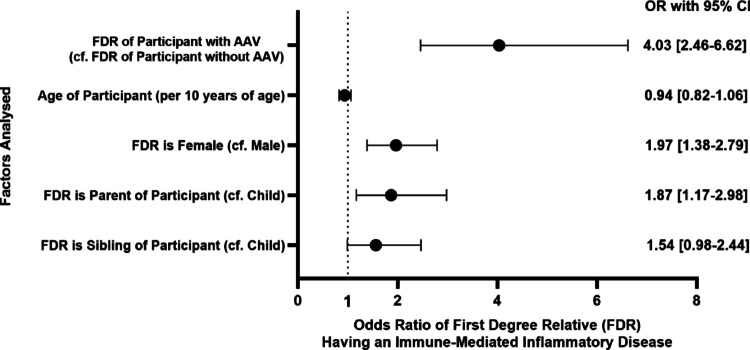
Factors associated with first-degree relatives having immune-mediated inflammatory disease. AAV = ANCA-associated vasculitis; FDR = first-degree relative

Figure 3 presents the results of the secondary outcome analysis with the individual *first-degree relatives* treated as the observed cohort, rather than AAV patients or controls. First-degree relatives of a case (participants with AAV) had a four-fold increase in the likelihood of having immune-mediated inflammatory disease compared to first-degree relatives of controls (participants without AAV) (OR 4.03, 95% C.I. 2.46–6.62).

Additionally, we considered the influence of three other factors on the risk of individual first-degree relatives having immune-mediated inflammatory disease, regardless of the presence of AAV family history. The generational relationship of the first-degree relative to the participant was found to have an impact, with parents of participants having an increased risk when using children of participants as the reference group (OR 1.87, 95% C.I. 1.17–2.98). Another factor that increased the risk was the first-degree relative being female (OR 1.97, 95% C.I. 1.38–2.79). The age of the participant did not impact upon the risk (OR 0.94, 95% C.I. 0.82–1.06).

The increased risk of the cases’ first-degree relatives having immune-mediated inflammatory disease was sustained when additional analyses were performed for first-degree relatives, separated by their generational relationships to the participant. The cases’ parents, siblings and children had increased ORs of 3.95 (95% C.I. 2.04–7.64), 4.16 (95% C.I. 1.99–8.67) and 3.01 (95% C.I. 1.08–8.37) respectively when compared to the corresponding relatives of control participants (Supplementary Figure S2).

## Discussion

This study demonstrates a strong association between the presence of AAV and other immune-mediated inflammatory diseases in their first-degree relatives. AAV participants had more than a 7-fold increased likelihood of having a positive family history of immune-mediated inflammatory disease and conversely, a first-degree relative of an AAV participant had a more than 4-fold increased likelihood of having an immune-mediated inflammatory disease. These findings imply a shared genetic pre-disposition between AAV and other immune-mediated inflammatory diseases.

There has only been only other study which has investigated immune-mediated inflammatory diseases in first-degree relatives of people with GPA, using a population-based cohort study [14]. Knight et al. analysed Swedish health databases and performed data-linkage between datasets for GPA patients and their first-degree relatives. The study found a moderate increase of immune-mediated inflammatory disease in first-degree relatives of GPA patients compared with controls with a hazard ratio (HR) of 1.32, and found an increased risk with specific conditions, including multiple sclerosis (HR 1.92), Sjögren’s syndrome (HR 2.00), and seropositive rheumatoid arthritis (HR 1.54). Our results, using a different methodology, are concordant with the study’s conclusion of a shared genetic pre-disposition between GPA and other immune-mediated inflammatory diseases. However, there was a difference in the magnitude of findings, likely stemming from differences in study design. Knight et al.’s study uses a Swedish national health database, for which there is no equivalent in Australia, while our study used direct patient-reporting. Both approaches have their strengths and weaknesses. Whilst patient-reporting can be biased by the accuracy of patient recall, there can be difficulties with data linkage studies including under recording, linkage errors, incomplete data and potential misclassification [25]. Furthermore, Knight et al. relies on outpatient specialist review over a limited timeframe of six years to capture the diagnosis of immune-mediated inflammatory diseases in first-degree relatives. This means that first-degree relatives with immune-mediated inflammatory diseases may not be included if they did not receive specialist management during this period, potentially resulting in an underestimation of cases. Furthermore, Knight et al. considered only GPA patients and used a relatively restricted list of coded diseases. By comparison, our study included both GPA and MPA patients and included a wider range of immune-mediated inflammatory diseases reported. These reports were scrutinised where necessary in a blinded manner by a clinical immunologist not involved with the management of the participants.

Our study describes a familial relationship between AAV and a range of immune-mediated inflammatory diseases, with the higher frequency of several disease types in first-degree relatives of AAV participants, including musculoskeletal/connective tissue, endocrine, cutaneous, gastrointestinal and vasculitis type diseases. For less frequent types such as neurological, haematological and kidney diseases, the smaller number of first-degree relatives affected did not allow a meaningful comparison. Overall, our results suggest a consistent familial pre-disposition of AAV to a broad range of immune-mediated inflammatory disease types.

Only one AAV participant had a first-degree relative with AAV, compared to many first-degree relatives with different immune-mediated inflammatory diseases. These findings are concordant with those of Knight et al. whose use of a Swedish nationwide registry found familial clustering of GPA to be rare, with no pronounced familial risk amongst first-degree relatives [26]. Though there are a few case reports of rare monogenic disorders causing AAV, it overall lacks clear Mendelian inheritance [2]. The heritable component of AAV is likely to be polygenic, with a shared genetic pre-disposition to immune-mediated inflammatory diseases [2, 27].

While the current studies do not address mechanisms of the links between AAV and other immune-mediated inflammatory diseases, published studies have linked the risk of developing AAV with polymorphisms in several genes participating in common immunological pathways [6–9, 28–38]. For example, *PTPN22*, *CTLA-4* and *BACH2*, which influence T cell activity, have associations with AAV as well as other immune-mediated inflammatory diseases. Polymorphisms in PTPN22 have been associated with conditions including type-1 diabetes mellitus, rheumatoid arthritis, systemic lupus erythematosus and giant cell arteritis and one variant of CTLA-4 has been linked to Graves’ disease [6–9, 28–31]. BACH2 has been associated with immune-mediated inflammatory disease that include Hashimoto thyroiditis, Grave’s disease, type-1 diabetes mellitus, Crohn’s disease, coeliac disease, multiple sclerosis and vitiligo [32–37]. Additionally, monogenic diseases of the immune system have been documented to lead to autoimmunity and autoinflammation, including diseases affecting CTLA-4 and BACH-2 which impact regulatory T-cells [39]. These associations suggest shared genetic mechanisms that promote immune-mediated inflammatory diseases and are also shared with AAV [38].

Some studies have suggested that HLA class I-related (‘MHC-I-opathies’) and HLA-class-II-related diseases cluster among themselves [40]. AAV itself is associated with HLA class II alleles [2, 4, 6]. In our study, although there were a higher number of first-degree relatives of AAV cases with HLA class II related immune diseases including rheumatoid arthritis, systemic lupus erythematous and pernicious anaemia, there was also more relatives with the HLA-class-I linked disease psoriasis. However, the numbers affected are too small to confidently link AAV with other diseases linked to HLA class I or class II. Overall, our findings are consistent with studies in other immune-mediated inflammatory conditions including systemic lupus erythematous, multiple sclerosis, Sjogren’s Syndrome and Type 1 diabetes mellitus, that have shown familial clustering of immune-mediated inflammatory disease and imply the presence of common genetic mechanisms [11, 41–43].

Our study found a difference in the likelihood of AAV participants having a positive family history when separating the three different generational relationships of first-degree relatives. Disease presence in the parents of participants gave the highest odds ratio of participants having AAV, with siblings also having an increased odds ratio. The presence of disease in children yielded an odds ratio that did not reach significance, likely due to younger generations having less exposure time to immune-mediated inflammatory conditions. This difference was also demonstrated in the secondary outcome when first-degree relatives were analysed as a cohort. In the combined analysis, there was an increased likelihood for parents of participants to have immune-mediated inflammatory disease when directly compared to children.

Analyses investigating the risk of first-degree relatives having immune-mediated inflammatory disease identified an increased likelihood if the first-degree relative was female. It is well recognised that autoimmune conditions are more common in females with a prevalence double that of males, so as one would have expected, the study found female first-degree relatives had more immune-mediated inflammatory diseases at a similarly increased frequency [44].

This study has several strengths. First, almost all eligible AAV participants (98.0%) from our large tertiary healthcare network with a dedicated vasculitis clinic responded, reducing sampling and non-response bias due to the near complete description of first-degree relatives in our AAV patient cohort. Second, our study used an appropriate control group with chronic health issues (nephrology patients without immune-mediated inflammatory diseases), who are more likely to have knowledge of their medical history and the medical conditions of their first-degree relatives, compared to a healthy population. The AAV patient cohort also had a high proportion of kidney involvement (72%), which makes nephrology patients a relevant matched control group. However, those with AAV, most of whom would understand that this is an immune-mediated disease, may have more awareness of these diseases. This may lead to recall bias due to having more detailed knowledge of whether their family members also suffered from an immune-mediated disease.

The current study has some limitations. The main limitation is the requirement of participants to directly report immune-mediated inflammatory diseases in relatives, with the absence of clinical confirmation of these diseases by a clinician. This raises the risk of recall bias, such as the misclassification of diseases and differences in the reporting between cases and controls. To minimise this bias, participants were provided with a list of medical conditions for reference, and the control group had chronic health issues with regular interaction with healthcare. As participants did not record the age of first-degree relatives, we could not determine their “exposure time” of developing an immune-mediated inflammatory disease. However, to accommodate this uncertainty, further analyses were performed separating generations of first-degree relatives as a proxy for age differences.

In conclusion, a history of immune-mediated inflammatory disease within the family increases the risk of a person developing AAV. Conversely, the presence of AAV in a first-degree relative increases an individual’s chance of having an immune-mediated inflammatory disease. This suggests a shared susceptibility between AAV and other immune-mediated inflammatory diseases within families, providing support for studies that demonstrate their shared genetic associations with real-world clinical data.

## Supplementary Information

Below is the link to the electronic supplementary material.

Supplementary Material 1

## Declarations

### Writing of manuscript

We declare that artificial intelligence has not been used to write or revise this manuscript. We declare that external commercial editing support has not been used to write or revise this manuscript. No part of this report is copied or published elsewhere.

### Presentation of this research

This research was presented at the 22nd International Vasculitis Workshop in 2026 with an abstract published in the Zenodo Repository. The full citation of the workshop abstract publication is: Kitching, A. R., Hill, C., & Quincey, V. (2026). Proceedings of the 22nd International Vasculitis Workshop, Melbourne, Feb 22–25, 2026. Proceedings of the 22nd International Vasculitis Workshop, 2026, 1–552. 10.5281/zenodo.18693153. This research was also presented at the Australian and New Zealand Society of Nephrology Congress in 2025, with an abstract published in Nephrology. The full citation of the congress abstract publication is: 2025 “Mini Orals.” Nephrology 30, no. S3: 24–65. 10.1111/nep.70089.
