# Supplementary material for: Familial associations between ANCA-associated vasculitis and other immune-mediated inflammatory diseases: a case control study
Source: Rheumatol Int. 2026 Jul 24;46(8):221. doi: 10.1007/s00296-026-06259-1 (PMC13400474; doi:10.1007/s00296-026-06259-1)
Supplement: Supplementary file 1 — Supplementary Material 1 [file 296_2026_6259_MOESM1_ESM.docx]

Supplementary Information

**Familial associations between ANCA-associated vasculitis and other immune-mediated inflammatory diseases: a case control study**

**Journal:** Rheumatology International

**Authors:**

Justin Chong Meng CHUA

Kevan Roy POLKINGHORNE

Samar OJAIMI

Jessica RYAN

Arthur Richard KITCHING

**Corresponding author:**

**Justin Chong Meng CHUA**

Department of Medicine, Monash University, Clayton, Victoria, Australia

Address: Monash University Department of Medicine, Monash Medical Centre, 246 Clayton Rd, Clayton, VIC 3168, Australia.

Email: [justin.chua@monash.edu](mailto:justin.chua@monash.edu)

**List of Supplementary Information**

Supplementary Figure S1: List of Immune-Mediated Inflammatory Diseases Provided to Participants for their Reference

Supplementary Table S1: Strengthening the Reporting of Observational Studies in Epidemiology (STROBE) Guidelines for Case Control Studies Checklist

Supplementary Table S2: Immune-Mediated Inflammatory Diseases Amongst AAV Cases and First-Degree Relatives of Participants

Supplementary Figure S2: Risk of First-Degree Relatives of AAV Cases having Immune-Mediated Inflammatory Disease by Generational Relationship to Participant

**Supplementary Figure S1: List of Immune Mediated Inflammatory Diseases Provided to Participants for their Reference**

| **List of** **Immune Mediated Inflammatory Diseases**  ***Groups and Specific Diseases*** |
| --- |

This is a list of immune-mediated inflammatory disease groups, which may or may not be applicable to you and your family members. Under each group are examples of these diseases. *However, this is not a complete list – you may tell us about other immune diseases that are not listed.*

A number of immune-mediated inflammatory diseases can fall into more than one group. Please select the one you think is most relevant.

Please look through the list to gain some understanding of the conditions.

- **Autoimmune vasculitis – *inflammation of blood vessels***
  - This condition includes diseases called:
    - ANCA (anti-neutrophil cytoplasmic antibody) associated vasculitis
      - Wegener’s granulomatosis / Granulomatosis with polyangiitis
      - Churg-Strauss / Eosinophilic granulomatosis with polyangiitis
      - Microscopic polyangiitis (MPA)
    - Henoch Schonlein Purpura (HSP)
    - Temporal arteritis
- **Autoimmune / inflammatory connective tissue disease (including joints)** **– *includes inflammation of joints (hands, wrists, knees etc.) and back***
  - This condition includes diseases called:
    - Rheumatoid arthritis
    - Systemic lupus erythematous (lupus)
    - Scleroderma
    - Ankylosing spondylitis
    - Polymyalgia rheumatica
    - Polymyositis/dermatomyositis
    - Mixed connective tissue disease
    - Sjogren’s syndrome
    - Sarcoidosis/ other granulomatous disease
- **Autoimmune / inflammatory kidney disease – *includes inflammation of small blood vessels of kidneys (called glomerulonephritis)***
  - This condition includes diseases called:
    - IgA nephropathy
    - Goodpasture’s syndrome
    - Membranous nephropathy
    - Minimal change disease
    - Lupus nephritis
- **Autoimmune / inflammatory neurological condition – *includes inflammation of brain, nerves or spinal cord***
  - This condition includes diseases called:
    - Multiple sclerosis (MS)
    - Guillain-Barre Syndrome
    - Myasthenia gravis
    - Chronic inflammatory demyelinating polyneuropathy (CIDP)
    - Acute disseminated encephalomyelitis (ADEM)
    - Autoimmune encephalopathies
- **Autoimmune endocrine (hormone) disease – *disease of an endocrine gland that secretes a hormone***
  - This condition includes diseases called:
    - Type 1 diabetes mellitus – *diabetes which usually, but not always, happens at a younger age and needs injected insulin therapy at the time of diagnosis*
    - Autoimmune thyroid disease
      - Grave's disease (hyperthyroidism – overactive thyroid)
      - Hashimoto's disease (hypothyroidism – underactive thyroid)
- **Autoimmune / inflammatory gastrointestinal disease**
  - This condition includes diseases called:
    - Inflammatory bowel disease (Crohn's, Ulcerative colitis)
    - Coeliac disease
    - Primary biliary cirrhosis
    - Primary sclerosing cholangitis
    - Autoimmune hepatitis
- **Autoimmune / inflammatory skin diseases** - *skin conditions considered auto-immune*
  - This condition includes diseases called:
    - Vitiligo
    - Psoriasis
    - Pemphigoid
    - Pemphigus
    - Alopecia areata
- **Immune haematological (blood) and bone marrow disorders**
  - This condition includes diseases called:
    - Immune thrombocytopenic purpura (ITP)
    - Pernicious anaemia
    - Anti-phospholipid syndrome
- **Immunodeficiency (primary)** – ***defect in immune system often due to genetic reasons contributing to recurrent infections****. This does NOT include immunodeficiency due to other reasons such as infection (human immunodeficiency virus [HIV]) or medications (chemotherapy, immunosuppressive medications etc.)*
- **Other immune diseases**

**Supplementary Table S1 – STROBE Statement—Checklist of items that should be included in reports of case-control studies**

*Page numbers according to location in manuscript*

|  | Item No | Recommendation | Page No |
| --- | --- | --- | --- |
| **Title and abstract** | 1 | (*a*) Indicate the study’s design with a commonly used term in the title or the abstract | 1 |
|  |  | (*b*) Provide in the abstract an informative and balanced summary of what was done and what was found | 4 (of title page) |
| Introduction | | | |
| Background/rationale | 2 | Explain the scientific background and rationale for the investigation being reported | 1 |
| Objectives | 3 | State specific objectives, including any prespecified hypotheses | 1 |
| Methods | | | |
| Study design | 4 | Present key elements of study design early in the paper | 2 |
| Setting | 5 | Describe the setting, locations, and relevant dates, including periods of recruitment, exposure, follow-up, and data collection | 2-3 |
| Participants | 6 | (*a*) Give the eligibility criteria, and the sources and methods of case ascertainment and control selection. Give the rationale for the choice of cases and controls | 2 |
|  |  | (*b*) For matched studies, give matching criteria and the number of controls per case | 2 |
| Variables | 7 | Clearly define all outcomes, exposures, predictors, potential confounders, and effect modifiers. Give diagnostic criteria, if applicable | 3 |
| Data sources/ measurement | 8* | For each variable of interest, give sources of data and details of methods of assessment (measurement). Describe comparability of assessment methods if there is more than one group | 3 |
| Bias | 9 | Describe any efforts to address potential sources of bias | 3 |
| Study size | 10 | Explain how the study size was arrived at | 4-5 |
| Quantitative variables | 11 | Explain how quantitative variables were handled in the analyses. If applicable, describe which groupings were chosen and why | 3-4 |
| Statistical methods | 12 | (*a*) Describe all statistical methods, including those used to control for confounding | 4 |
|  |  | (*b*) Describe any methods used to examine subgroups and interactions | 4 |
|  |  | (*c*) Explain how missing data were addressed | 3 |
|  |  | (*d*) If applicable, explain how matching of cases and controls was addressed | 3 |
|  |  | (*e*) Describe any sensitivity analyses | N/A |
| Results | | | |
| Participants | 13* | (a) Report numbers of individuals at each stage of study—eg numbers potentially eligible, examined for eligibility, confirmed eligible, included in the study, completing follow-up, and analysed | 6-7 |
|  |  | (b) Give reasons for non-participation at each stage | 6-7 |
|  |  | (c) Consider use of a flow diagram | 7 |
| Descriptive data | 14* | (a) Give characteristics of study participants (eg demographic, clinical, social) and information on exposures and potential confounders | 8-9 |
|  |  | (b) Indicate number of participants with missing data for each variable of interest | 8-9 |
| Outcome data | 15* | Report numbers in each exposure category, or summary measures of exposure | 10 |

| Main results | | 16 | (*a*) Give unadjusted estimates and, if applicable, confounder-adjusted estimates and their precision (eg, 95% confidence interval). Make clear which confounders were adjusted for and why they were included | 10-15 |
| --- | --- | --- | --- | --- |
|  |  |  | (*b*) Report category boundaries when continuous variables were categorized | 10-15 |
|  |  |  | (*c*) If relevant, consider translating estimates of relative risk into absolute risk for a meaningful time period | N/A |
| Other analyses | 17 | Report other analyses done—eg analyses of subgroups and interactions, and sensitivity analyses | | 14-15 |
| Discussion | | | | |
| Key results | 18 | Summarise key results with reference to study objectives | | 16-17 |
| Limitations | 19 | Discuss limitations of the study, taking into account sources of potential bias or imprecision. Discuss both direction and magnitude of any potential bias | | 18-19 |
| Interpretation | 20 | Give a cautious overall interpretation of results considering objectives, limitations, multiplicity of analyses, results from similar studies, and other relevant evidence | | 17-19 |
| Generalisability | 21 | Discuss the generalisability (external validity) of the study results | | 16-19 |
| Other information | | | | |
| Funding | 22 | Give the source of funding and the role of the funders for the present study and, if applicable, for the original study on which the present article is based | | 2 (of title page) |

*Give information separately for cases and controls.

**Note:** An Explanation and Elaboration article discusses each checklist item and gives methodological background and published examples of transparent reporting. The STROBE checklist is best used in conjunction with this article (freely available on the Web sites of PLoS Medicine at http://www.plosmedicine.org/, Annals of Internal Medicine at http://www.annals.org/, and Epidemiology at http://www.epidem.com/). Information on the STROBE Initiative is available at http://www.strobe-statement.org.

**Supplementary Table S2:** **Immune-Mediated Inflammatory Diseases Amongst AAV Cases and First-Degree Relatives of Participants**

|  | *Number Reported* | | |
| --- | --- | --- | --- |
| **Type of Immune-Mediated Inflammatory Disease** | **AAV Patient**  **(Cases)** | ***First Degree Relative* of Cases (AAV)** | ***First Degree Relative* of Controls** |
|  |  |  |  |
| ***Musculoskeletal and Connective Tissue*** |  |  |  |
| Rheumatoid Arthritis | 4 | 27 | 5 |
| Systemic Lupus Erythematous | 4 | 5 | 1 |
| Inflammatory Arthritis | 0 | 3 | 1 |
| Psoriatic Arthritis | 0 | 1 | 0 |
| Ankylosing Spondylitis | 0 | 1 | 0 |
| Scleroderma | 0 | 1 | 0 |
| Still’s Disease | 0 | 1 | 0 |
| Juvenile Rheumatoid Arthritis | 1 | 0 | 0 |
| Mixed Connective Tissue Disease | 1 | 0 | 0 |
| ***Endocrine*** |  |  |  |
| Hyperthyroidism (incl. Grave’s) | 5 | 13 | 2 |
| Hypothyroidism (incl. Hashimoto’s) | 11 | 12 | 4 |
| Thyroid Issues (Not Specified) | 0 | 3 | 1 |
| Type 1 Diabetes Mellitus | 4 | 9 | 2 |
| Addison’s Disease | 0 | 0 | 1 |
| ***Cutaneous*** |  |  |  |
| Psoriasis | 6 | 12 | 3 |
| Vitiligo | 2 | 1 | 0 |
| Alopecia | 0 | 1 | 0 |
| Lichen Planus | 0 | 1 | 0 |
| Sweet’s Syndrome | 2 | 0 | 0 |
| Bullous Pemphigoid | 1 | 0 | 0 |
| Lichen Sclerosus | 1 | 0 | 0 |
| ***Gastrointestinal*** |  |  |  |
| Coeliac Disease | 3 | 4 | 1 |
| Inflammatory Bowel Disease | 1 | 4 | 1 |
| Pernicious Anaemia | 2 | 2 | 0 |
| Autoimmune Hepatitis | 1 | 0 | 0 |
| ***Vasculitis*** |  |  |  |
| Giant Cell Arteritis / PMR | 0 | 4 | 1 |
| ANCA-Associated Vasculitis | N/A | 1 | N/A |
| Kawasaki’s Disease | 0 | 1 | 0 |
| Vasculitis (Not Specified) | 0 | 1 | 0 |
| Henoch Schonlein Purpura | 1 | 0 | 0 |
| Goodpasture’s Syndrome (anti-GBM) | 1 | 0 | 0 |
| ***Neurological*** |  |  |  |
| Multiple Sclerosis | 0 | 2 | 4 |
| Guillain Barre Syndrome | 0 | 1 | 0 |
| ***Haematological*** |  |  |  |
| Immune Thrombocytopenia Purpura | 1 | 1 | 1 |
| Primary Immunodeficiency | 0 | 1 | 1 |
| Anti-Phospholipid Syndrome | 0 | 1 | 0 |
| Neutropenia | 0 | 0 | 1 |
| Thrombotic Thrombocytopenic Purpura | 0 | 0 | 1 |
| ***Kidney*** |  |  |  |
| Nephritis (Bright’s Disease) | 0 | 1 | 0 |
| Nephrotic Syndrome | 0 | 0 | 1 |
| ***Other Immune*** |  |  |  |
| Sarcoidosis | 0 | 2 | 0 |
| Non-Specific Autoimmune Disease | 0 | 1 | 0 |
| Sjogren’s | 1 | 0 | 0 |

There are first-degree relatives of cases and controls who have more than one immune-mediated inflammatory disease, which are counted separately in the table.

**Supplementary Figure S2:** **Risk of First-Degree Relatives of AAV Cases having** of **Immune-Mediated Inflammatory Diseases by Generational Relationship to Participant**


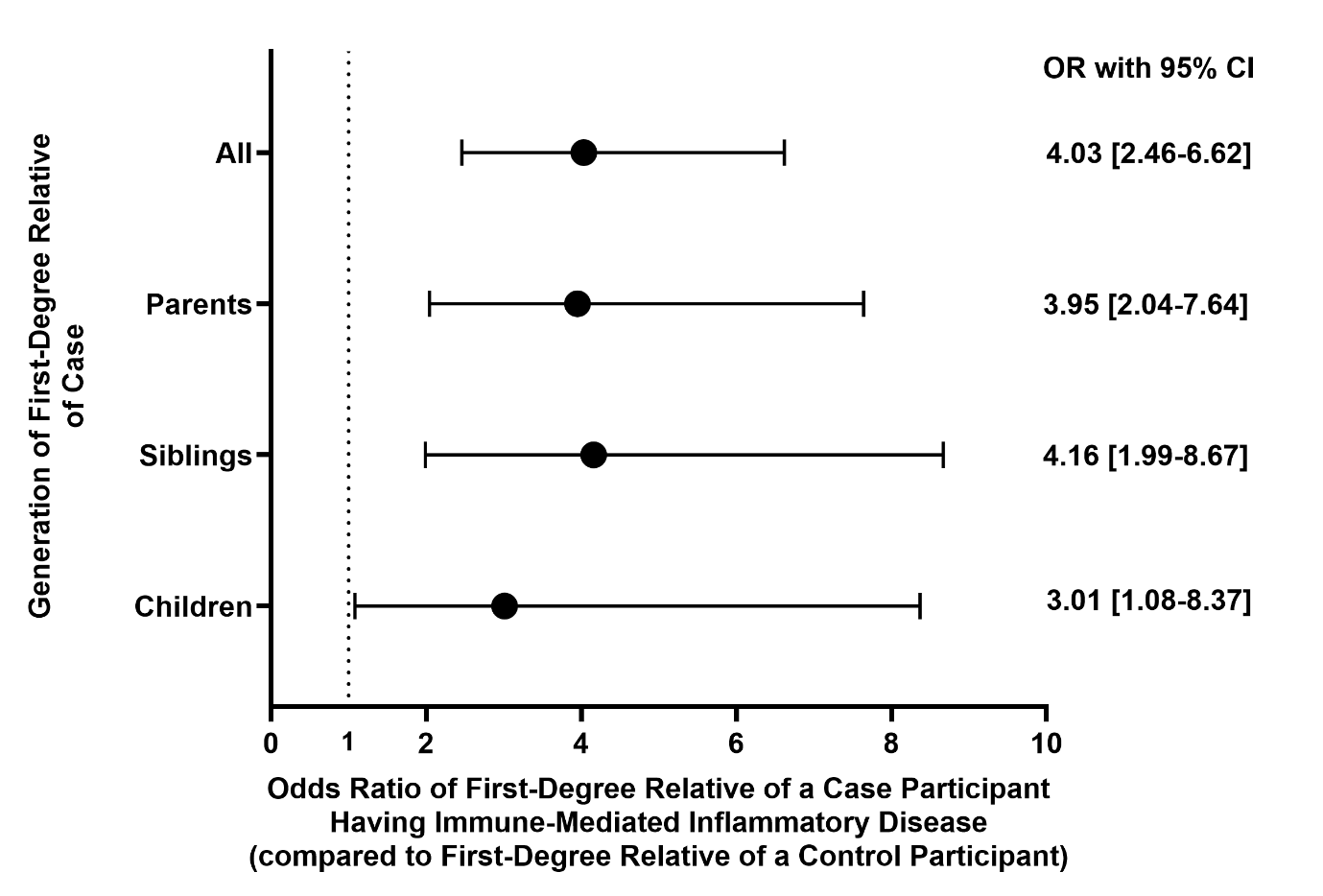


AAV = ANCA-associated vasculitis
